# Can Preoperative Examination Help Choose the Best Surgical Procedure in Gastric Cancer?

**DOI:** 10.1155/2018/4914201

**Published:** 2018-04-01

**Authors:** Jia-Le Zhang, Zhen-Ning Wang, Hui-Mian Xu, Zhi Zhu, Bao-Jun Huang

**Affiliations:** Department of Surgical Oncology, First Affiliated Hospital, China Medical University, Shenyang, Liaoning, China

## Abstract

**Aim:**

Gastrectomy with lymph node dissection is standard treatment in gastric cancer. This study aimed to explore whether preoperative investigation finds could predict lymph node metastatic scope in gastric carcinoma so that the optimal surgical procedure could be selected.

**Materials and Methods:**

Radical gastrectomy patients (*n* = 378) were separated into two groups according to the lymph node metastatic scope. Univariate and multivariate analyses of preoperative examination results were performed to identify the predictors of metastatic scope. ROC curves were constructed, and the area under the curve (AUC) was calculated to estimate diagnostic values.

**Results:**

Serum CEA (OR: 3.73; 95% CI: 1.84–7.56; *P* ≤ 0.001), tumor size (OR: 2.07; 95% CI: 1.08–3.98; *P* = 0.03), and CT examination results (OR: 17.81; 95% CI: 9.18–34.55; *P* ≤ 0.001) were identified as independent predictors. The AUC proved that they possessed significant diagnostic value. When CT examination was negative, the combination of serum CEA and tumor size showed high specificity (95.3%; 164/172), negative predictive value (92.7%; 164/177), and accuracy (89.0%; 170/191).

**Conclusions:**

Preoperative serum CEA, tumor size, and CT examination are independent predictors of lymph node metastatic scope and can be used for selecting the appropriate lymphadenectomy pattern in gastric cancer patients.

## 1. Introduction

Although the mortality of gastric cancer is declining as a result of the tremendous advances in therapeutic methods, it is still the second most common cause of cancer-related death in China and the world [1–3]. Recurrence and metastasis are the major threats to gastric cancer patients [4, 5]. Of all the metastatic patterns, lymphatic metastasis is most common. It is also an independent prognostic factor [6, 7], and therefore, special attention must be paid to lymphatic metastasis in gastric cancer patients.

Gastrectomy with lymph node dissection remains the first choice and standard treatment for the majority of gastric cancer patients. According to the Japanese Gastric Cancer Treatment Guidelines 2010 [8], only early gastric cancer (EGC) with differentiated histologic type or no lymph node metastasis is suitable for D1 and D1+ lymphadenectomy. Other EGC and advanced gastric cancer patients require D2 lymphadenectomy. However, this therapeutic strategy is too general. Every patient has unique clinicopathological features, and the therapeutic approach should be personalized to avoid the problem of over- and underdissection of lymph nodes.

Theoretically, the choice of the lymphadenectomy pattern must be strictly based on the metastatic scope. But, in fact, the status of lymph node metastasis cannot be accurately established even by pathological examination after operation, which is the gold standard test, let alone by other investigation results. A research from Japan, involving 929 patients, has reported that multidetector row CT (MDCT) could predict the preoperative N staging for resectable cT2–4 gastric carcinoma [9]. But only a few studies have focused on the metastatic scope of lymph nodes. We hypothesized that the results of preoperative investigations such as esophagogastroduodenoscopy (EGD), computed tomography (CT), and serum tumor marker levels could be used to roughly predict the metastatic scope, and on this basis, the best pattern of lymphadenectomy could be chosen for each patient. In this study, we present a novel concept, that is, to use preoperative examination results to formulate the treatment strategy, especially the extent of lymphadenectomy.

## 2. Materials and Methods

### 2.1. Data Sources and Study Population

The study population included 378 patients who underwent radical gastrectomy with standard D2 lymphadenectomy (the proximal with D1+) between January 1, 2011, and December 31, 2012, at the Department of Surgical Oncology, First Affiliated Hospital of China Medical University. Among these patients, 71 had EGCs. All patients had histologically proven gastric carcinoma and had undergone radical (R0) resection. No patient had malignant tumor of other sites or distant metastasis. None of the patients had received any neoadjuvant therapies. The number of lymph nodes retrieved were all no less than 16 based on the lowest standard of the UICC/AJCC TNM staging system [10].

The range of lymphadenectomy in different types of gastrectomies has been clearly defined in the Japanese Gastric Cancer Treatment Guidelines 2010 (ver. 3) [8] (Table 1). Whether in distal, total, or proximal gastrectomy, the resection scope of D1 is always limited to the perigastric lymph nodes. With D1+ and D2 lymphadenectomy, the scope is extended to the second and third stations. All patients in this study had received standard D2 lymphadenectomy; we classified those patients whose lymph nodes had metastasized to the extent of D1+ and D2 as the “beyond perigastric” group, and the remainder whose metastatic scope was limited to D1 or who had no lymph node metastasis were classified as the “perigastric” group.

Whether the lymph nodes were positive for metastasis was checked by at least 2 pathologists independently. The diagnosis was confirmed by a more experienced pathology specialist. Data on histological type, Borrmann type, tumor size, and tumor location were all collected by esophagogastroduodenoscopy before surgery, and the findings were confirmed by pathology specialists. All patients received 3-D enhanced-CT examination of the stomach within 1 week before surgery, and the reports were verified by radiologists. Venous blood collected on the first morning after hospitalization was tested for hemoglobin (HGB), white blood cell (WBC) count, lymphocyte count, granulocyte (GRAN), and blood platelet (PLT). The collected venous blood was centrifuged at 3000 rpm for 5 minutes, and the serum was analyzed for tumor markers such as CEA, CA199, AFP, CA125, and CA153 using a fully automatic electrochemistry luminescence immunity analyzer (Roche cobas e 411; Roche Diagnostics, Mannheim, Germany).

**Figure 1:**
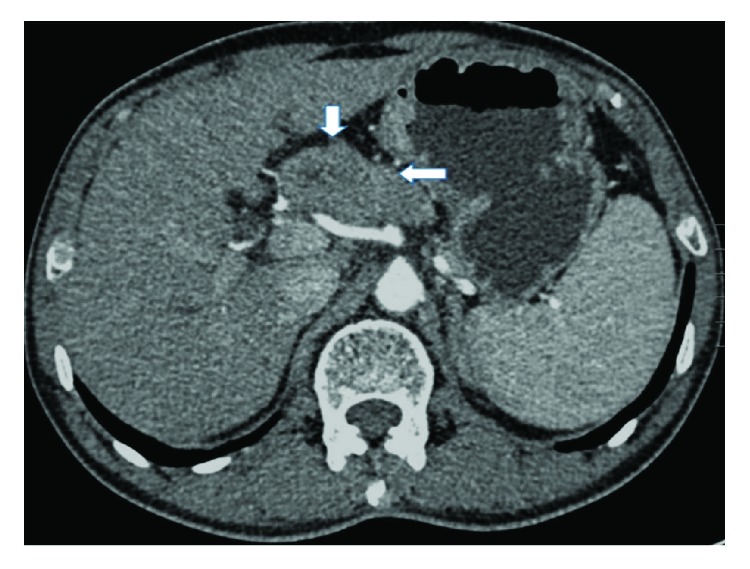
CT image obtained in a 64-year-old man. A big lymph node with a short-axis diameter > 8 mm can be seen along the lesser curvature of the stomach.

**Figure 2:**
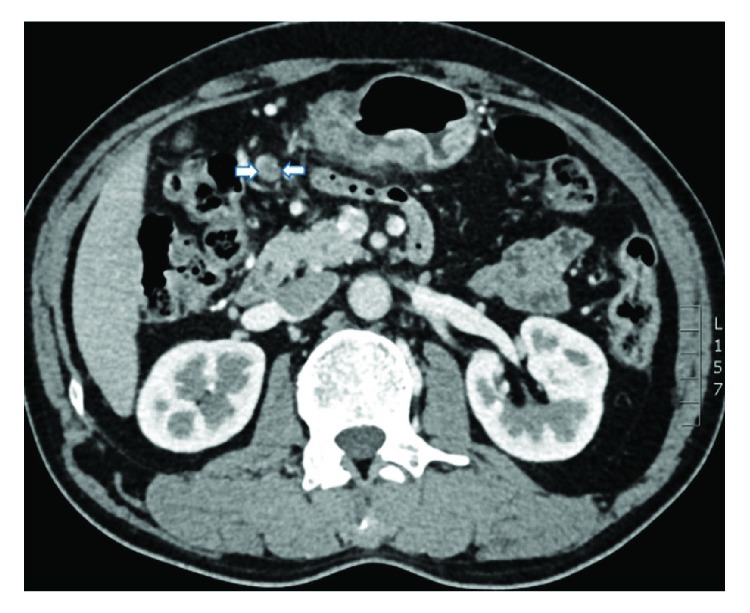
CT image obtained in a 54-year-old man. A round-shaped lymph node with a central low-attenuation area is visualized beside the right gastric artery.

**Figure 3:**
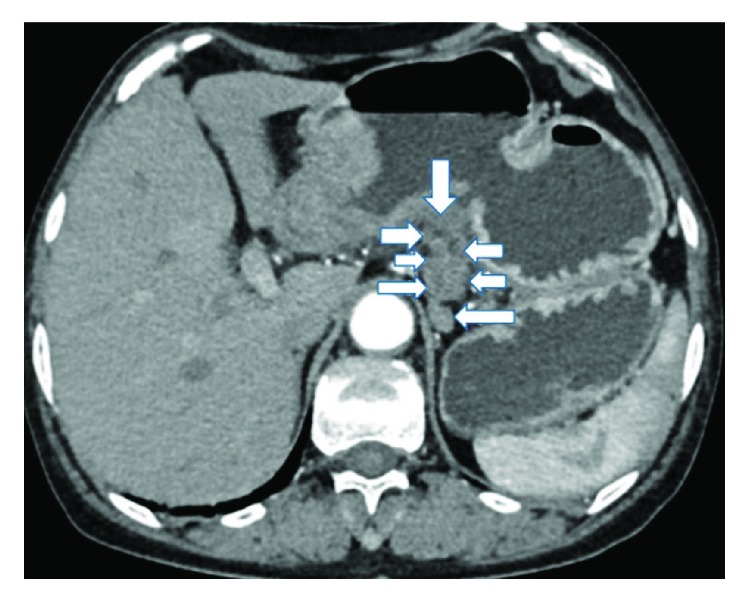
CT image obtained in a 65-year-old woman. Clustered lymph nodes (>6) can be seen along the lesser curvature of the stomach.

**Figure 4:**
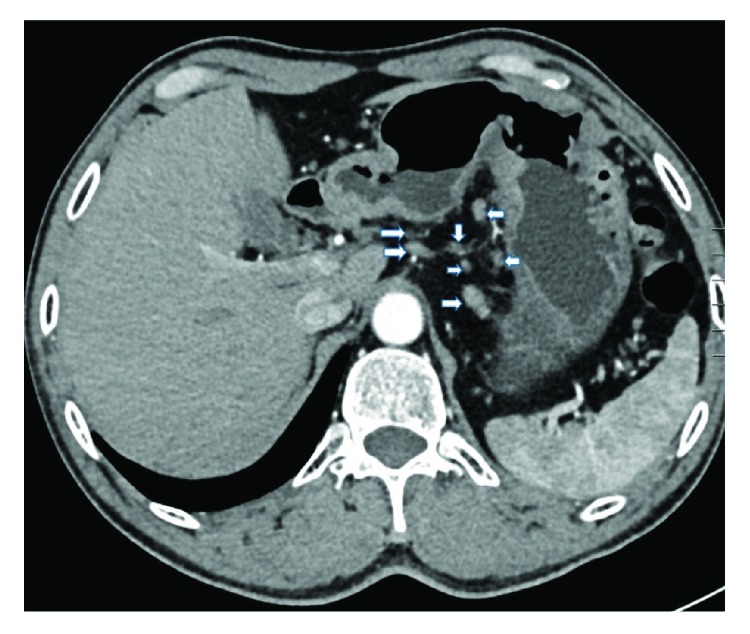
CT image obtained in a 50-year-old man. Isolated but multiple (>7) lymph nodes are seen around the lesser curvature of the stomach.

**Figure 5:**
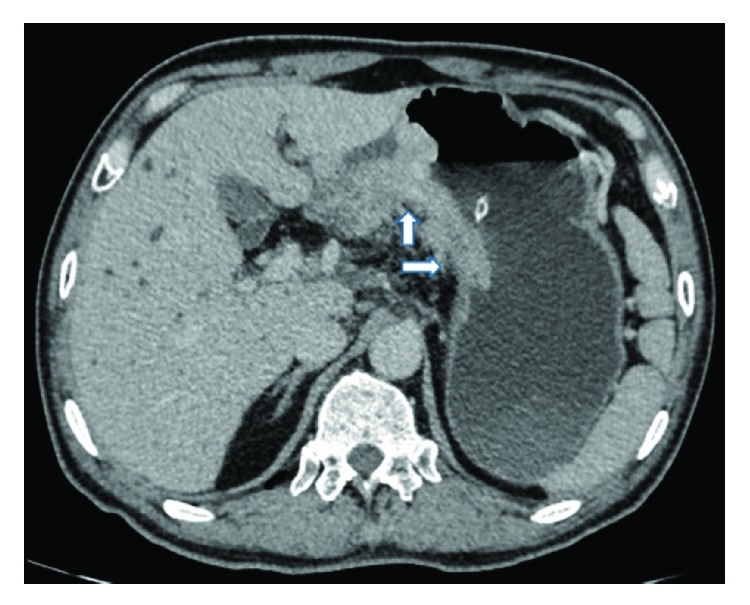
CT image obtained in a 56-year-old man. The serosal surface is rough around the lesser curvature of the stomach.

**Figure 6:**
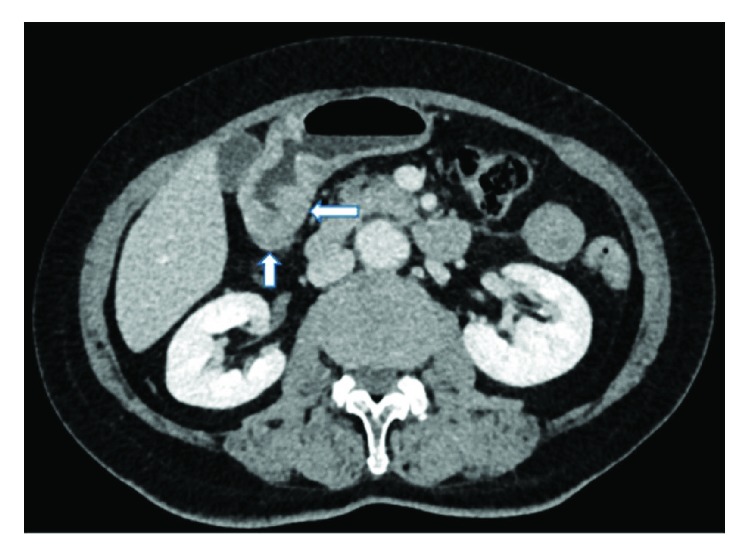
CT image obtained in a 75-year-old woman. The serosal surface is smooth around the lower 1/3of the stomach.

A radiologist and an experienced gastric surgeon, who were both blinded to the endoscopic findings and the medical history of the patient, analyzed the 3-D enhanced-CT images of the abdomen to predict the lymphatic metastatic scope and cT stage. If there was any disagreement, the final diagnosis was arrived at by discussion. Lymph nodes were diagnosed as positive if (1) they were enlarged, with the shortest axis diameter being ≥8 mm; (2) they were circular in shape, with a central low-density area, suggesting necrosis; or (3) there was clustering of ≥3 nodes or (4) they were isolated but multiple, that is, >5 nodes (Figures 1–4) [11–13]. All patients in whom such nodes were identified were thought to have lymph node metastasis. As for the cT stage, it is hard to make a precise judgement of the cT1, cT2, and cT3 stages by CT scan reported by previous studies [14–16]; as a result, we divided these patients into the cT1–cT3 group (tumor invasion restricted to the subserous layer) and the cT4 group (tumor invasion beyond serosa). Patients should be included in the cT4 group when the serosal surface was rough according to the CT scan (Figure 5). On the contrary, patients should be included in the cT1–cT3 group when the serosal surface was smooth according to the CT scan (Figure 6) [14–16].

All preoperative examination results, including HGB, WBC count, lymphocyte count, granulocyte count, serum tumor markers, Borrmann type, tumor size, tumor location, tumor histologic type, cT stage, and outcome of 3-D enhanced-CT examination of the abdomen, were compared between the perigastric group and the beyond perigastric group. Among the different histologic types, papillary cancers and tubular cancers were classified as the differentiated type, while mucinous cancers, signet-ring cell cancers, poorly differentiated cancers, and undifferentiated cancers were grouped together as the undifferentiated type. Among the Borrmann types, types 1 and 2 (with clear margins) were classified as the localizable group, while Borrmann types 3 and 4 (without definite margins) were classified as the infiltrative group [17].

### 2.2. Statistical Analysis

Statistical analysis was carried out using SPSS 17.0 statistical package (SPSS Inc., Chicago, IL, USA). In univariate analysis, the 2-tailed chi-square test and 2-tailed *t*-test were used for the comparisons of categorical variables and continuous variables, respectively. The parameters identified as significant on the univariate analysis were subjected to multivariate analysis. ROC curves were drawn to assess the diagnostic value of those parameters that were proved to be independent risk factors by multivariate analysis. *P* < 0.05 was considered statistically significant. Area under the curve (AUC) > 0.5 and *P* ≤ 0.05 indicated a statistically significant finding. The best cutoff value of serum CEA was estimated from the ROC curve.

The (independent and combined) sensitivity, specificity, positive predictive value (PPV), negative predictive value (NPV), and accuracy of the significant parameters were calculated, and ROC curves were used to identify the best method to predict the scope of lymph node metastasis.

## 3. Results

### 3.1. Outcomes of Univariate and Multivariate Analyses

The preoperative investigation results of the two groups are shown in Table 2. Univariate analysis showed significant differences between the 2 groups in HGB (*P* = 0.007), lymphocyte count (*P* = 0.009), serum CA19-9 (*P* = 0.04), serum CEA (*P* ≤ 0.001), CT examination findings (*P* ≤ 0.001), cT stage (*P* ≤ 0.001), tumor size (*P* ≤ 0.001), Borrmann type (*P* = 0.03), and differentiated type (*P* ≤ 0.001) (Table 3).

On multivariate analysis (Table 3), serum CEA (OR: 3.73; 95% CI: 1.84–7.56; *P* ≤ 0.001), tumor size (OR: 2.07; 95% CI: 1.08–3.98; *P* = 0.03), and CT examination (OR: 17.81; 95% CI: 9.18–34.55; *P* ≤ 0.001) were found to be independent predictors of the scope of lymph node metastasis.

### 3.2. Comparison of Diagnostic Values in Different Situations

**Figure 7:**
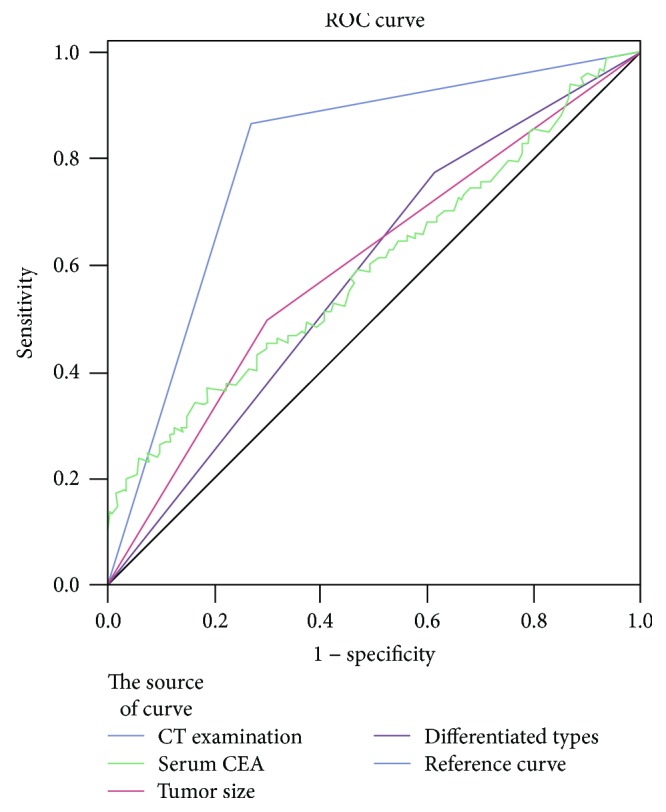
The ROC curves of the independent risk factors identified by multivariate analysis.

The ROC curves of the independent predictors are shown in Figure 7. The AUC was greatest for CT examination (AUC = 0.80; *P* ≤ 0.001). The AUC for serum CEA was 0.60 (*P* = 0.002) and that for tumor size 0.60 (*P* ≤ 0.001). Thus, all the three parameters showed significant predictive ability. In addition, we identified 3.37 ng/mL as the best cutoff value of serum CEA.

After identifying the independent predictors of lymphatic metastatic scope, we calculated their sensitivity, specificity, positive predictive value (PPV), negative predictive value (NPV), and accuracy when used independently and in combination (Table 4). CT examination possessed the highest sensitivity (86.5%; 122/141) and accuracy (78.0%; 295/378); its specificity was also not low at 73.0% (173/237).

Having identified CT examination as the most valuable independent diagnostic method, we investigated the value of the other two independent predictors when CT examination was negative and positive (Tables 5 and 6). When CT examination was negative, the combination of serum CEA and tumor size showed high specificity (95.3%; 164/172), NPV (92.7%; 164/177), and accuracy (89.0%; 170/191). However, when CT examination was positive, serum CEA and tumor size showed no additional predictive value.

## 4. Discussion

According to the Japanese Gastric Cancer Treatment Guidelines 2010 (ver. 3) [8], standard gastrectomy involves resection of at least two-thirds of the stomach along with a D2 lymph node dissection. It is the principal surgical procedure performed with curative intent. All advanced gastric cancers and EGCs with lymph node metastasis should undergo standard gastrectomy [8]. However, the outcome with this treatment approach is controversial. One study from the US that enrolled 727 patients found that D2 lymphadenectomy could improve survival rates and should be considered for gastric adenocarcinoma patients [18]. On the other hand, two other studies (one from Japan and another from the Netherlands) reported that extended lymph node dissection provided no survival benefit, as any advantage in long-term survival was offset by the higher postoperative mortality [19, 20]. Consequently, D2 lymphadenectomy cannot be considered the optimal choice for all advanced gastric cancers and EGCs with lymph node metastasis as stipulated in the Japanese Gastric Cancer Treatment Guidelines 2010. For patients whose metastatic scope is limited to D1 and those without lymph node metastasis, D2 lymphadenectomy seems to be inappropriate. Thus, it would be useful if preoperative examination results could be used to predict the lymph node metastatic scope in gastric cancer patients.

In the current study, gastric cancer patients who underwent D2 lymphadenectomy were separated into a beyond perigastric group and a perigastric group according to their metastatic scope, and the results of preoperative examinations in these two groups were compared. Serum CEA, CT examination, and tumor size were confirmed as independent predictors of metastatic scope.

Ychou et al. [21] have reported that CEA functions as a homotypic intercellular adhesion molecule, and so cells expressing this glycoprotein may have greater invasive potential. Other studies have certified that in gastric cancer patients with liver metastasis, the serum CEA level is always distinctly elevated [22, 23]. This can explain why a high level of serum CEA is an independent risk factor for lymph node metastasis scope. Previous studies have also reported that in both early and advanced gastric cancers, patients with tumor size > 3 cm have higher probability of lymph node metastasis than those with tumors of <3 cm size [24–26]. We also found that tumor size > 5 cm was an independent predictor of lymph node metastatic scope, while for lymphocyte count, it was found to be a significant predictor of lymph node metastatic scope in univariate analysis but not in multivariate analysis. Based on the findings of previous studies, the lymphocyte count is associated with prognosis of gastric cancer patients and can be used in early diagnosis [27–29], while the relationship between lymphocyte count and lymph node metastasis (especially the metastatic scope) was rarely studied. More researches still need to be carried out to explore the relationship between lymphocyte count and lymph node metastasis. In the future, we will focus on this kind of research.

It is worth mentioning the value of the differentiation type (histologic types), the Borrmann type, and cT stage. Previous studies have reported that in EGCs, the differentiation type is an independent risk factor for lymph node metastasis [30–32]. In our research, it was found to be significant on univariate analysis but was excluded as an independent predictor by multivariate analysis. Tong et al. [33] found that in EGCs, irrespective of clinicopathologic features or prognosis, signet-ring cell cancer was similar to differentiated type cancer in behavior. Our sample contained both advanced and early gastric cancer patients. Therefore, it was unreasonable to consider all signet-ring cell cancer as a poorly differentiated type. The relationship between the signet-ring cell cancer and differentiated type cancer need further research, especially in advanced gastric cancer. Therefore, this factor was not further investigated in our research.

Li et al. [34] have reported that the Borrmann types 1 and 2 had no significant difference in prognosis (*P* = 0.55). In terms of depth of invasion (*P* = 0.49), lymph node involvement (*P* = 0.14), hepatic metastasis (*P* = 0.53), peritoneal dissemination (*P* = 0.49), and TNM stage (*P* = 0.16), the two types were similar. However, when compared with Borrmann types 3 and 4, significant differences were found in prognosis (*P* ≤ 0.001) and clinicopathologic features. For lymph node metastasis, the Borrmann type was shown to be an independent risk factor (*P* < 0.05) in advanced gastric cancer. These results demonstrate that the infiltrative type (including Borrmann types 3 and 4) have greater invasive ability, and they therefore tended to be grouped in the beyond perigastric group. Similar to the differentiation type, the Borrmann type was found to be a significant predictor of lymph node metastatic scope in univariate analysis but not in multivariate analysis. The reason could be limitations in comprehensiveness, visibility, and accuracy of esophagogastroduodenoscopy performed in the clinic. Additionally, not all cancer foci have typical shapes. Because of these two reasons, there is much scope for misdiagnosis when determining the Borrmann type. There is therefore a need for the development of more precise and comprehensive examination equipment.

As for the cT stage, it is accepted that the cT stage from CT examination may be related to lymph node metastasis. Like other preoperative examinations, cT stage was found to be significant on univariate analysis but was excluded as an independent predictor by multivariate analysis. Due to the improvement of equipment and technology of multislice spiral CT, the improvement of gastric filling status, the application of dynamic contrast-enhanced intravenous injection, and the application of three-dimensional reconstruction of CT images, the evaluation and judgement of the cT stage has been improved [14–16]. However, there are still some limitations, especially in the judgement of cT1, cT2, and cT3. As a result, we could only divide all the gastric cancer patients into the cT1–cT3 group and the cT4 group by the serosal surface. From my point of view, the limitation of CT in diagnosing the cT1, cT2, and cT3 stages and the inevitable mistakes in the judgement of whether the serosal surface is rough or smooth may reduce the accuracy and application value of CT. And we hope we can overcome such shortcomings in our future studies.

Analysis of ROC curves for the three independent predictors identified by multivariate analysis revealed that all three had significant and comparable diagnostic value (AUC > 0.5; *P* < 0.05). For clinical application, we identified the ideal cutoff value of serum CEA to be 3.37 ng/L.

We examined the diagnostic value of the three independent predictors, both when used independently and in combination. As shown in Table 4, of the three, CT examination had the highest sensitivity, specificity, and accuracy (86.5% [122/141], 73.0% [173/237], and 78.0% [295/378], resp.). It also had the highest NPV of 89.6% (173/193). We found that when CT examination was negative, the specificity and NPV of the combination of serum CEA and tumor size both exceeded 90%; however, this effect was not seen when the CT examination was positive.

In our study, the beyond perigastric group included those with the metastatic lymph nodes in stations which required D2 lymphadenectomy for resection (Table 1). For these patients, the D2 lymphadenectomy is no doubt the best option. On the other hand, as shown in Table 1, no matter in distal or total gastrectomy, the group 12a was only contained in D2 lymphadenectomy while 8a was in D1+ and D2. The 12a patients were those with hepatoduodenal ligament lymph nodes along the proper hepatic artery, in the caudal half between the confluence of the right and left hepatic ducts and the upper border of the pancreas. Meanwhile, the 8a patients were those with anterosuperior lymph nodes along the common hepatic artery [17]. However, these two groups were adjacent actually. It is quite difficult to clearly distinguish them during operation or during retrieval of lymph nodes after operation. In order to avoid missing the metastatic lymph nodes, especially 12a, D2 lymphadenectomy should be used in these patients even though their metastatic scope is limited to D1+.

According to our data, the sensitivity of CT examination was 86.5% (122/141) but the PPV was only 65.5% (122/185). This means that if we use CT examination in predicting the metastatic scope based on the above four criteria, 34.5% (63/185) of the gastric cancer patients in our sample who were classified as “beyond perigastric” should actually have been classified as “perigastric.” Although our findings await confirmation in larger studies, it appears that there is sufficient reason to adopt D2 lymphadenectomy in patients with positive CT examination. On the current situation, the 3-D enhanced-CT examination for stomach can reluctantly be used as a criterion in the clinic.

As we have shown, when the CT examination is negative, the combination of serum CEA and tumor size can be used to predict the metastatic scope with a fair degree of certainty. However, when CT examination is negative and serum CEA and tumor size are below the cutoff, one can be reasonably certain that the patient either has lymph node metastasis limited to D1 scope or has no lymph node metastasis. In these patients, therefore, D1 lymphadenectomy is the most appropriate choice, irrespective of whether they have EGC or advanced disease. Thus, using our findings, it will be possible to avoid unnecessary D2 lymphadenectomy in some patients.

However, when applying this method, care must be taken to ensure the accuracy of all results. Despite the availability of advanced equipment and the latest reagents, errors are possible. Tumor size, tumor site, Borrmann type, and histologic type of the focus are of crucial importance both before and after surgery. Endoscopy and biopsy are excellent methods for preoperative diagnosis, but accuracy of interpretation must be ensured. Last but not least, the surgical team should do their best to resect the available lymph nodes during operation to achieve R0 resection. The procedure of retrieving lymph nodes from the resected specimen after operation is also very important and must be performed diligently, especially with regard to recording the number of nodes and the specific stations, which is vital for staging and prognostication.

Our study has certain limitations. First, this was a retrospective study, where the results were based on postoperative examination of resected specimens and retrieved lymph nodes. Human error is unavoidable in such situations, especially when retrieving metastatic lymph nodes from a resected specimen. Second, we followed the present classification of histological types and included signet-cell cancer under undifferentiated cancers; this classification is however controversial. Signet-cell cancer is similar to differentiated cancers in many respects. Signet-ring cell cancers also have a lower incidence of lymph node metastasis and a more favorable prognosis than other undifferentiated cancers [35, 36]. This classification may have biased our results to some extent. Third, the small sample size of this study restricts the quality of the result.

In the future, we will set a training and external or internal validation cohorts to detect the value of these preoperative investigations.

## 5. Conclusions

In this study we identified CT examination, serum CEA, and tumor size as independent predictors of lymph node metastatic scope in gastric cancer patients. When CT examination is negative, the combination of serum CEA and tumor size can be used to predict the metastatic scope and help minimize the degree of lymph node dissection. CT examination has high sensitivity and accuracy as an independent predictor of the metastatic scope and therefore can be used as a criterion for adopting D2 lymphadenectomy. A training and external or internal validation cohorts should be set to detect the value of these preoperative investigations.

## Figures and Tables

**Table 1:** The number and range of lymphadenectomy in different gastrectomies.

Gastronomy	Number	The range of lymphadenectomy		
		D1	D1+	D2
Total	145	1–7	D1+ numbers 8a, 9, and 11p	D1+ numbers 8a, 9, 10, 11p, 11d, and 12a
Distal	201	Numbers 1, 3, 4sb, 4d, 5, 6, and 7	D1+ numbers 8a and 9	D1+ numbers 8a, 9, 11p, and 12a
Proximal	32	Numbers 1, 2, 3a, 4sa, 4sb, and 7	D1+ numbers 8a, 9, and 11p	

**Table 2:** Preoperative examination results of the beyond perigastric group and the perigastric group.

	Beyond perigastric	Perigastric
HGB (g/L)	123.11 ± 2.21	130.44 ± 1.64
GRAN (∗10^9^/L)	3.68 ± 0.15	3.63 ± 0.09
Lymphocytes (∗10^9^/L)	2.23 ± 0.19	1.78 ± 0.13
WBC (∗10^9^/L)	6.21 ± 0.17	6.31 ± 0.13
PLT (∗10^9^/L)	242.67 ± 5.42	233.54 ± 4.91
CA19-9 (U/mL)	64.61 ± 15.01	30.54 ± 6.74
CEA (ng/mL)	11.15 ± 2.35	2.48 ± 0.18
AFP (U/mL)	10.84 ± 6.41	5.10 ± 1.97
CA12-5 (U/mL)	14.06 ± 0.87	14.65 ± 1.23
CA15-3 (U/mL)	8.67 ± 0.46	9.32 ± 0.51
Age	58.26 ± 0.87	58.94 ± 0.69
Sex		
Male	98	177
Female	43	60
CT examination		
Positive	173	19
Negative	63	123
cT stage		
cT1–cT3	44	84
cT4	110	140
Tumor site		
Upper 1/3	15	29
Middle 1/3	20	54
Lower 1/3	100	150
Occupy more than 2 areas	6	4
Tumor size		
>5 cm	70	70
<5 cm	71	167
Differentiated types		
Undifferentiated type	109	144
Differentiated type	32	93
Borrmann types		
Localizable group	9	25
Infiltrative group	128	145

**Table 3:** Univariate and multivariate analysis for all the gastric cancer patients.

	Univariate analysis		Multivariate analysis		
	*c* ^2^(*t*)	*P*	RR	95% CI	*P*
Borrmann type	5.10	0.03			
CEA	−4.75	≤0.001	3.73	1.84–7.59	≤0.001
CA19-9	−2.06	0.04			
CT examination	126.43	≤0.001	17.81	9.18–34.56	≤0.001
cT stage	11.04	≤0.001			
HGB	2.69	0.007			
Lymphocytes	2.01	0.009			
Differentiated types	10.93	≤0.001			
Tumor size	15.56	≤0.001	2.07	1.08–3.98	0.03

**Table 4:** Diagnostic value of the predictors of lymph node metastatic scope when used independently and in combination.

Group	Sensitivity (%)	Specificity (%)	PPV (%)	NPV (%)	Accuracy (%)
CEA	37.9	81.1	54.1	68.9	65.08
CT examination	86.5	73.0	65.9	89.6	78.00
Tumor size	49.6	70.6	50.0	70.3	62.70
CEA and CT examination	17.0	96.2	72.7	66.2	66.75
CEA and tumor size	28.4	94.1	74.1	68.9	69.66
Tumor size and CT examination	43.3	84.4	62.2	71.4	69.05
All the three combined	33.3	87.8	61.8	69.0	67.60

**Table 5:** Diagnostic value of tumor size and serum CEA when CT examination was negative.

Group	Sensitivity (%)	Specificity (%)	PPV (%)	NPV (%)	Accuracy (%)
Tumor size	57.9	72.8	19.0	94.0	71.35
CEA	47.4	80.9	21.4	93.3	77.60
Tumor size and CEA	31.6	95.3	42.9	92.7	89.00

**Table 6:** Diagnostic value of tumor size and serum CEA when CT examination was positive.

Group	Sensitivity (%)	Specificity (%)	PPV (%)	NPV (%)	Accuracy (%)
Tumor size	48.4	65.1	72.8	39.4	54.05
CEA	35.8	82.5	79.6	40.3	51.91
Tumor size and CEA	69.2	52.4	73.5	47.1	63.39
